# Outcome of interventions to improve the quality of intrapartum care in Nigeria’s referral hospitals: a quasi-experimental research design

**DOI:** 10.1186/s12884-023-05893-y

**Published:** 2023-08-26

**Authors:** Friday Okonofua, Lorretta Favour Ntoimo, Bola Ekezue, Victor Ohenhen, Kingsley Agholor, Wilson Imongan, Rosemary Ogu, Hadiza Galadanci

**Affiliations:** 1 Centre Leader, Centre of Excellence in Reproductive Health Innovation, University of Benin, Benin City, Nigeria; 2 Department of Obstetrics and Gynaecology, University of Benin, University of Benin Teaching Hospital, Benin City, Nigeria; 3 Women’s Health and Action Research Centre (WHARC), Benin City, Nigeria; 4 Department of Demography and Social Statistics, Federal University Oye-Ekiti, Ekiti, Nigeria; 5 Fayetteville State University, Fayetteville, USA; 6 Department of Obstetrics and Gynaecology, Central Hospital Benin City, Benin City, Nigeria; 7 Department of Obstetrics and Gynaecology/Anti-Retroviral Therapy Centre, Central Hospital, Warri, Delta State Nigeria; 8 Department of Obstetrics and Gynaecology, University of Port Harcourt, Choba, Rivers State Nigeria; 9 Department of Obstetrics and Gynaecology, Bayero University, Kano, Nigeria

**Keywords:** Intrapartum care, Midwives, Exit interviews, Nigeria hospitals, Maternal mortality, Quality of care, Maternal satisfaction

## Abstract

**Background:**

Evidence indicates that Nigeria’s high maternal mortality rate is attributable primarily to events that occur during the intrapartum period. This study determines the effectiveness of multifaceted interventions in improving the quality of intrapartum care in Nigeria’s referral hospitals.

**Methods:**

Data collected through an exit interview with 752 women who received intrapartum care in intervention and control hospitals were analyzed. The interventions were designed to improve the quality indicators in the WHO recommendations for positive childbirth and assessed using 12 quality indicators. Univariate, bivariate, Poisson, and logistic regression analyses were used to compare twelve quality indicators at intervention and control hospitals.

**Results:**

The interventions showed a 6% increase in composite score of quality of care indicators at intervention compared with control hospitals. Five signal functions of intrapartum care assessed were significantly (< 0.001) better at intervention hospitals. Quality scores for segments of intervention periods compared to baseline were higher at intervention than in control hospitals.

**Conclusions:**

We conclude that multiple interventions that address various components of the quality of intrapartum care in Nigeria’s referral hospitals have demonstrated effectiveness. The interventions improved five of ten quality indicators. We believe that this approach to developing interventions based on formative research is important, but a process of integrating the implementation activities with the normal maternal health delivery processes in the hospitals will enhance the effectiveness of this approach.

**Trial registration:**

The study was registered at the Nigeria Clinical Trials Registry. Trial Registration Number NCTR No: 91,540,209 (14/04/2016) http://www.nctr.nhrec.net/ and retrospectively with the ISRCTN. Trial Registration Number 64 ISRCTN17985403 (14/08/2020) 10.1186/ISRCTN17985403.

## Background

Available evidence indicates that Nigeria’s high maternal mortality rate is largely attributable to events that occur during the intrapartum (delivery) period [1–3]. To date, only 43.3% of pregnant Nigerian women are attended to at birth by skilled birth attendants (midwives, nurses, and medical doctors)[4]. By contrast, the Nigeria Demographic and Health Survey data indicate that 0.5–71.8% of pregnant women depending on the location in the country, utilize unskilled traditional birth attendants [4].

The reasons proffered by women for use of unskilled birth attendants in Nigeria are multiple [5, 6] but they mostly center on their lack of satisfaction with the intrapartum and delivery care they receive in orthodox health facilities [7, 8]. We, therefore, hypothesize that when women are satisfied with intrapartum care, it would lead to a multiplier effect in increasing women’s use of skilled intrapartum care within community settings.

The World Health Organization has made a series of recommendations for optimal intrapartum care for a positive birth experience [9]. Some of the recommendations based on consensus and research include uterine tonus assessment, pain control, hemorrhagic disease prevention using vitamin K, intrapartum breastfeeding, skin-to-skin contact, delayed umbilical cord clamping, and controlled cord traction.

To date, there has been limited documentation of formative assessment of the quality of intrapartum care [10], and very little is known of how women are attended to in labour in the country as well as the level of satisfaction or dissatisfaction with intrapartum care [7]. Also not known is the level to which health facilities have implemented interventions that address improved quality of care in labor and the impact of such interventions on the outcomes of labor.

It was within this context that the maternal health research team at the Women’s Health and Action Research Centre, a non-governmental, and non-profit organization in collaboration with partners across the country, first conducted a series of qualitative and quantitative research to understand the factors associated with the intrapartum care in eight hospitals from four of the six geo-political zones in Nigeria [11]. We also undertook qualitative research with women to determine their experiences of intrapartum care in the hospitals, and the factors they associated with positive and negative experiences [8]. We then used the results to design a series of interventions that address women’s concerns in two hospitals, while two other hospitals with similar characteristics located in the same zones served as the non-intervention control hospitals.

The objective of this study was to determine the effectiveness of the multifaceted intervention in improving the quality of intrapartum care as reported in exit interviews conducted in intervention as compared to the control hospital. We believe the results will be useful in informing the design and implementation of policies and practices for improving the quality of intrapartum care and increasing the use of skilled birth attendants in the country.

## Methods

### Study Design and setting

The study was drawn from a larger quasi-experimental research conducted in referral hospitals in Nigeria with the broad goal of improving the quality of obstetric care [8, 12–14]. The outcome indicators in the larger research included reduction in incidence and improved management of post-partum haemorrhage, eclampsia, obstructed labour, improvement in antenatal, intrapartum, and postnatal care, among others [15–18]. There was a mixed-method formative research stage to explore diverse dimensions of obstetric care in Nigeria, intervention and post intervention stages. Eight secondary health facilities participated in the formative research, and four went through to the intervention stage of the research. The current analysis presents an assessment of the quality of intrapartum care in the four secondary hospitals: two intervention hospitals and two control hospitals in the South-South, and the North-Central regions of Nigeria. The Central Hospital, Benin City, and General Hospital, Minna as intervention hospitals, and Central Hospital, Warri, and General Hospital, Suleja, as control hospitals. Nigeria operates a three-tier health system that has primary health care as the first tier. The second tier consists of the secondary hospitals also called general hospitals and the teaching hospitals at the third tertiary level. The secondary and tertiary hospitals are referral hospitals that offer both basic and comprehensive obstetric care.

### Ethical approval

Ethical approval for the study was obtained from the World Health Organization and the National Health Research Ethics Committee (NHREC) of Nigeria with registration number NHREC/01/01/2007 **(16/07/2014)**, renewed in 2015 with registration number NHREC 01/01/20047b **(12/12/2015).** Verbal consent to participate was approved by the ethics committee. The Chief Medical Directors, and Heads of Departments in the hospitals were informed of the purpose of the study, and verbal consent was obtained from them to conduct the study. Verbal informed consent was obtained from the participants. They were assured of the confidentiality of the information obtained. No names or specific contact information were obtained from the study participants. The research methods were performed in accordance with the Declaration of Helsinki on research involving human participants.

### Intervention design and implementation

Following the formative study, the results were presented at meetings with healthcare providers in the intervention hospitals and used to design and plan the interventions. The interventions consisted of (1) strategic visioning and plan to improve intrapartum and delivery care; (2) re-training of maternal health providers to improve delivery care; (3) provision of protocols, algorithms and reminders on optimal intrapartum care; (4) clinical reviews of delivery care; and (5) health education provided to pregnant women. The details of the intervention activities are described below:

*Strategic planning for improved delivery care*. We used findings from the formative research to inform the SWOT analysis that determined the strengths, weaknesses, opportunities, and threats in providing optimal intrapartum care in the hospitals. We then identified the challenges that needed to be addressed and developed a strategic plan for implementing the intervention activities. The Medical Directors of the hospitals then worked with a team (committee) consisting of the Heads of the Obstetrics and Gynaecology Departments, the head midwives, and the Consultant obstetricians and gynaecologists in the hospitals to implement the activities identified in the plan.

The intervention hospitals were re-designated as “women-friendly hospitals” to provide the best experiences to women during pregnancy, labour, and post-natal care.

*Re-training of maternal health workers*. The training was based on the deficits in intrapartum clinical management identified during the formative research and focused on: the mechanism and physiology of labour, partographic monitoring of labour, active management of second and third stages of labour, management of complications of all phases of labour, the use and avoidance of episiotomy when not needed, pain relief in labour, management of the baby at birth, and appropriate ways to interact with women in labour. The training was for health workers in the obstetrics and gynaecology department and was facilitated by experienced obstetricians/midwives clinical experts and consisted of lectures, clinical demonstrations, role-plays, and discussions. Post-lecture compared to pre-lecture questions evaluations showed increased knowledge scores attained by participants at the end of the workshop.

*Development of management protocols, algorithms, and reminders.* As part of the staff re-training, protocols and algorithms for the management of labour were co-developed with all participants and printed thereafter for use in the labour wards of the hospital. Specifically, we re-developed the forms on parthographic monitoring of labour using the WHO guidelines and shared the specific WHO guidelines on labour management of labour with all staff. These included the interpretation of delays in labour, the use of drugs to initiate or augment labour, the indications and methods of caesarean section, the steps in decision-making and use of caesarean section, and the appropriate use of oxytocic drugs (oxytocin, ergometrine, and misoprostol) for the management of the third stage of labour and its complications. Additionally, we designed and posted reminders on labour management, partographic monitoring, pain relief in labour, and the use of drugs for second and third-stage labour management.

*Clinical reviews of delivery care and maternal mortality*. A daily review of the management of labour was commenced in the hospitals. Although the Central Hospital previously held daily meetings, we restructured the meetings to include reviews of all cases of labour management using the partograph, and to identify situations where the management protocols were breached. Daily meetings with all staff in the maternity and labour wards commenced to ensure knowledge sharing of procedures for the management of all stages of labour. During these periods, all maternal deaths and severe morbidities were reviewed by the clinical staff before submission to the hospitals’ maternal mortality reviews and surveillance committees [19].

*Health education and feedback from women*. As part of the intervention activities, weekly meetings were organized with pregnant women and their spouses. The meetings were organized on Saturdays to provide adequate time and opportunity for interactions between staff, pregnant women, and their caregivers. It consisted of presentations made by senior clinical care providers on various aspects of maternity care, followed by questions and answer sessions. Videos on procedures for admission of pregnant women into labour and during labour, including pain management, were shown. We also shared pictorial information on labour care during the meetings as well as an informational leaflet on frequently asked questions by pregnant women developed as part of the intervention.

### Data source

We conducted exit interviews with 752 women who received intrapartum care in four referral hospitals over 21 months, from October 2017 to June 2019. The baseline was the 1st–3rd month; 4th–9th month, intervention period; immediate post-intervention, 10th–15th month; and 16th–21st month was post-intervention period. The sample size for the exit interview was derived using the Yamane formula [20]. A sample size of 2400 was estimated, with 800 for each type of care (antenatal, intrapartum, and postnatal care). Of the 2262 interviews that were successfully conducted, 752 were for intrapartum care, 390 at the intervention hospitals and 362 at the control hospitals. Women were recruited for the interview if they gave birth to a baby at the intervention or the control hospitals during the study period, and consented to be interviewed. Data were collected every month from consenting women. The exit interview questionnaire was adapted from the World Bank, Federal Ministry of Health, and National Bureau of Statistics Health Results-Based Financing Nigeria 2017 Exit Interview questionnaire [21]. Socio-demographic data and information about the women’s experiences with some signal functions recommended by the WHO for optimal intrapartum care were obtained. The interviews were conducted at the time the women were being discharged from the hospitals, by trained women who did not participate in the clinical management of the patients. They were questioned confidentially to determine whether some signal functions recommended by the WHO for optimal intrapartum care were carried out when health workers attended to them.

The questions were organized in three sections. In section 1, information was solicited on the socio-demographic characteristics of the respondents – age, marital status, highest educational levels attained, religious, and occupational status. In the second section, we asked questions on the pregnancy history, whether they received antenatal, delivery and postnatal care in the indexed hospital or in other hospitals, and the nature of any pregnancy complication that may have experienced. In the final part of the questionnaire, we solicited information on specific aspects of their pregnancy and childbirth experiences.

### Variables and measures

The outcome of interest, quality of intrapartum care, was assessed using twelve quality indicators(QIs) selected from the WHO recommendations for positive childbirth experience [9]. See Table 2 for a list of the indicators and their distribution by site. The response options for most of the QIs were (Yes and No). Three questions had three response options (Yes, No, and Don’t know). No, and don’t know were merged into one category. The expected recommended response option was coded 1 and 0 otherwise. Other QIs without a yes and no response (provider who attended to the woman, mode of delivery, and duration before the first bath of the baby), were recoded based on WHO recommendation. The attending provider during delivery was recoded 0 for doctor only, and nurse/midwife only, and both doctor and nurse/midwife recoded 1. This is because WHO recommends both midwife/nurse/doctors be present for the Ritgen’s manoeuvre, perineal massage, warm perineal compresses, and upright birth positions, among others. Therefore, we considered the presence of a midwife/nurse/doctor as the ideal. The mode of delivery options were vaginal, emergency, and elective cesarean section (CS). The two types of CS were merged into a category and coded 0 whereas vaginal birth was coded 1. Duration before a baby’s first bath was categorized into < 24 h coded 0 and 24 + coded 1. The WHO recommends that the bathing of a baby should be delayed until 24 h after birth.

**Table 1:** Distribution of the study population by characteristics and study periods

Item	Both sites (N=752)	Control site (N = 362)	Intervention site (N = 390)	p-value
**Mean Age** (SD)	29.9(5.4)	30.4(4.9)	29.3(5.8)	0.0043
**Median number of children (IQR)**				
Range 0–10	2(2)	2(2)	2(2)	0.4199
	N(%	N(%)	N (%)	
**Period**				
Baseline	121(16.09)	61(16.85)	60(15.38)	0.585
Intervention	631(83.91)	301(83.15)	330(84.62)	
**Segmented period**				
Oct-Dec 2017 (Baseline)	121(16.09)	61(16.85)	60(15.38)	0.123
Jan-Mar 2018	121916.09)	60(16.57)	61(15.64)	
Apr-June 2018	97(12.90)	40(11.05)	57(14.62)	
July-Sept 2018	81(10.77)	30(8.29)	51(13.08)	
Oct-Dec 2018	109(14.49)	50(13.81)	59(15.13)	
Jan-Mar 2019	116(15.43)	61(16.85)	55(14.10)	
Apr-June 2019	107(14.23)	60(16.57)	47(12.05)	
**Age (grouped)**				
15–19	21(2.79)	5(1.38)	16(4.10)	0.005
20–24	96(12.77)	38(10.50)	58(14.87)	
25–29	232(30.85)	105(29.01)	127(32.56)	
30–34	244(32.45)	135(37.29)	109(27.95)	
35–39	126(16.76)	67(18.51)	59(15.13)	
40–49	33(4.39)	12(3.31)	21(5.38)	
**Highest education**				
No formal education	39(5.19)	10(2.76)	29(7.44)	0.035
Primary	78(10.37)	37(10.22)	41(10.51)	
Secondary	339(45.08)	166(45.86)	173(44.36)	
Higher	296(39.36)	149(41.16)	147(37.69)	
**Marital Status**				
Not in union	17(2.26)	9(2.49)	8(2.05)	0.689
In union	735(97.74)	353(97.51)	382(97.95)	
**Religion**				< 0.001
Catholic	109(14.49)	70(19.34)	39(10.00)	
Other Christian	460(61.17)	241(66.57)	219(56.15)	
Islam	183(24.34)	51(14.09)	132(33.85)	
**Occupation**				0.042
Not working	177(23.54)	78(21.55)	99(25.38)	
Civil servant	89(11.84)	33(9.12)	56(14.36)	
Self employed	432(57.45)	225(62.15)	207(53.08)	
Private sector employee	54(7.18)	26(7.18)	28(7.18)	
**Diagnosed of complication**				0.004
Yes	220(29.26)	124(34.25)	96(24.62)	
No	532(70.74)	238(65.75)	294(75.38)	

Ten QIs that involved all the respondents were aggregated to generate a single index of the quality of intrapartum care. A higher score indicates better quality of care. Two QIs (satisfaction with care during labour and prompt repair of episiotomy) were excluded from the aggregation because they were questions for a sub-group of the respondents.

#### Explanatory variables

The main explanatory variables were the site (intervention versus control site) and the research period (baseline and intervention). The intervention period was further segmented into seven periods of three months from baseline to the end of the intervention.

#### Control variables

Since the QI measures were based on the subjective report of the respondents, the effect of the personal characteristics of the respondents, which differed significantly between sites, was adjusted. These included age, the highest level of education, religion, occupation, and diagnosis of complications such as antepartum haemorrhage, and pre-eclampsia, among others. Age was in single years, but for the descriptive purpose, it was re- categorised into five-year groups except the last category (40–49) because of few cases in this bracket. The highest level of education was categorized as no formal education, primary, secondary and higher. Religion was categorized as Catholic, Other Christian, and Islam. The occupation of the respondents was grouped into not working, civil servant (government employee), self-employed, and private sector employee.

### Data analysis

All the data were collected using computer-assisted personal interview (CAPI) and extracted into Stata version 13 for analysis. The characteristics of the respondents for each arm were described using summary statistics, frequency, and percentage as applicable. The difference between sites was examined using a t-test or the non-parametric alternative for continuous variables, and the chi-square test of independence for categorical variables. The outcome variable was a count variable. Thus, the count of reporting the ten indicators was estimated using Poisson regression. The vce (robust) command in Stata was used to adjust for mild violation of the Poisson regression assumptions. Two models (unadjusted and adjusted) were estimated to compare the outcome between sites at baseline and intervention periods and within sites between the two periods. Individual-level factors that were significant at the bivariate level were added as control variables in the adjusted models. Hospital-level variable such as the provider who attended to the woman during labour and childbirth was not significantly different between the sites and period. Further analysis using logistic regression models was conducted for each of the ten QIs as an outcome and site as the independent variable in unadjusted and adjusted models for baseline and intervention periods. The overall average effect of the intervention was estimated with propensity score matching. All the regression analyses were two-tailed, and statistical significance was set at p < = 0.05.

## Results

The distribution of the respondents by socio-demographic characteristics and the study periods is presented in Table 1. The mean age of the respondents was 30.4 ± 4.9 in control, and 29.3 ± 5.8 in intervention hospitals. Most women were married and had an average of two children. Most participants in both sites attained secondary and higher education and were working. The majority were of the non-Catholic Christian denomination. Slightly above 70% were not diagnosed with any complications such as antepartum hemorrhage, or pre-eclampsia among others. The difference between site in the respondents’ age, highest education, religion, occupation, and diagnosed of complications was statistically significant.

**Table 2:** Quality Indicators of Intrapartum Care by Site

Quality Indicator	Control site (n = 362)	Intervention site (n = 390)	p-value
**Sum of 10 QIs mean(SD)**	6.9(1.4)	6.3(1.3)	
95% CI	6.7-7.0	6.2–6.4	< 0.001
	N (%)	N (%)	
**Delivery assistance**			0.910
Doctor only	23(6.35)	24(6.15)	
Nurse/Midwife/Both	339(93.65)	366(93.85)	
**Mode of delivery**			< 0.001
Caesarean section	126(34.81)	86(22.05)	
Vaginal	236(65.19)	304(77.95)	
**Clean delivery pack used**			< 0.001
No	3(0.83)	31(7.95)	
Yes	359(99.17)	359(92.05)	
**Anything applied on stump of umbilical cord**			< 0.001
No/don’t know	32(8.84)	194(49.74)	
Yes	330(91.16)	196(50.26)	
**Baby dried before placenta delivery**			0.527
No/don’t know	218(60.22)	226(57.95)	
Yes	144(39.78)	164(42.05)	
**Baby placed on belly/breast before placenta delivery**			0.890
No/don’t know	268(74.03)	287(73.59)	
Yes	94(25.97)	103(26.41)	
**Baby bathed first time**			< 0.001
< 24 hours	177(48.90)	96(24.62)	
24 hours +	185(51.10)	294(75.38)	
**Given verbal or printed information on baby care**			0.058
No	95(26.24)	127(32.56)	
Yes	267(73.76)	263(67.44)	
**Feel well attended during labour**			< 0.001
No	67(18.51)	35(8.97)	
Yes	295(81.49)	355(91.03)	
**Episiotomy**			0.268
Yes	99(27.35)	121(31.03)	
No	263(72.65)	269(68.97)	
**Sub-population**			
**Episiotomy repaired promptly**			< 0.001
No	33(33.33)	4(3.31)	
Yes	66(66.67)	117(96.69)	
**Experienced severe pain in labour**			< 0.001
Yes	234(64.64)	315(80.77)	
No	128(35.36)	75(19.23)	
**Happy with how health worker helped to reduce labour pain**			< 0.001
No	85(36.32)	52(16.51)	
Yes	149(63.68)	263(83.49)	

The distribution of the intrapartum care quality indicators is presented in Table 2. The mean scores of the quality indicators at intervention (6.3 ± 1.3) and control (6.9 ± 1.4) hospitals were significantly different. The percentage distribution of the responses to the quality indicators between site was statistically significant in five of the ten indicators. Most of the sub-group of respondents who had an episiotomy, almost 97% in the intervention sites, reported a prompt repair, and the difference between site is significant. About three-quarters of the respondents experienced severe pain in labour, and many were happy with how the health providers helped reduce labour pain. The difference between site was statistically significant.

Poisson regression was conducted to predict the count of intrapartum care scores(Table 3). At baseline (before the intervention), the count of intrapartum quality scores in the intervention site compared to the control site was higher in the unadjusted model, but decreased when other factors were adjusted and was not significant. During the intervention period, the count was significantly lower at intervention compared to the control sites in the unadjusted (IRR 0.89 CI: 0.86–0.92) and adjusted models (aIRR 0.87 CI: 0.85–0.90). The average treatment effect was also negative and significant.

**Table 3:** Poisson regression estimating the count of intrapartum care quality indicators between site and period

Variable	Baseline period		Intervention period		ATET(95% CI)
	IRR(95%CI)	aIRR(95% CI)	IRR(95%CI)	aIRR(95% CI)	
**Site**					-0.84
Control (Ref)					(-1.10- − 0.57)***
Intervention	1.03(0.95–1.13)	0.95(0.88–1.03)	0.89(0.86–0.92)***	0.87(0.85–0.90)***	
**Age**		1.00(0.99-1.00)		1.00(0.99-1.00)	
**Highest education**					
No formal education (Ref)					
Primary		1.10(0.95–1.29)		1.14(1.05–1.24)**	
Secondary		1.14(0.98–1.33)		1.08(1.00-1.16)*	
Higher		1.12(0.96–1.30)		1.04(0.96–1.13)	
**Religion**					
Catholic (Ref)					
Other Christian		0.98(0.93–1.03)		1.00(0.94–1.06)	
Islam		1.06(0.99–1.14)		1.04(0.97–1.11)	
**Occupation**					
Not working(Ref)					
Civil servant		1.02(0.94–1.11)		1.01(0.94-1.00)	
Self employed		0.97(0.92–1.03)		0.96(0.91–1.01)	
Private sector employee		0.97(0.89–1.05)		1.02(0.95–1.10)	
**Diagnosed of complication**					
Yes (Ref)		1.21(1.16-		1.14(1.08-	
No		1.26)***		1.20)***	

Note: IRR – Incidence Rate Ratio; aIRR – adjusted Incidence Rate Ratio; ATET – average treatment effect on the treated; *p < 0.05; **p < 0.01; ***p < 0.001

The control variables that were significant in adjusted models included education levels and any complications during labour. Women who attained primary, and secondary education compared to those who attained no formal education had significant higher count of QI indicators. The incidence rate ratio for women who reported no complications was higher compared to those who had complications, both at baseline and intervention.

Within site analysis (Table 4), comparing the count of intrapartum care quality at baseline and intervention revealed a statistically significant increase at the control site in both the adjusted and unadjusted models. The trend over the segmented periods in the adjusted model shows a statistically significant higher count of 20% during the intervention period, 14% during the immediate post-intervention and post intervention compared to the baseline. At the intervention site, the count of intrapartum care quality was 1.06 times greater during the intervention than at the baseline. Compared to the baseline, there was an insignificant lower incidence during the intervention, and an increase in count during the immediate post intervention periods, though statistically insignificant. In the post-intervention period, a a significant increase was observed(aIRR 1.06 CI:1.00-1.13) Estimating the count over the 21 months shows an insignificant increase in the control site over time when other factors are controlled. On the contrary, the increase in the experiment site was significant. The estimated average effect of the intervention on the treated during the intervention and immediate post-intervention periods were not significant, but it was significantly positive during the post intervention period.

**Table 4:** Poisson regression estimating the count of intrapartum care quality indicators within site over segmented period

Within site analysis - Change over segmented period					
	Control site		Intervention Site		
	IRR(95% CI) aIRR(95% CI)				ATET(95% CI)
Baseline (Ref)					
Intervention	1.23(1.15–1.32)***	1.20(1.12–1.27)***	1.06(1.00-1.12)*	1.06(1.00-1.11)*	0.49(0.21–0.77)**
Baseline (ref)					-0.38(-0.75- -0.02)*
Intervention period	1.20(1.12–1.30)***	1.20(1.12–1.28)***	0.96(0.90–1.02)	0.96(0.90–1.02)	-0.04(-0.34-0.25)
Immediate intervention period	1.16(1.08–1.26)***	1.14(1.06–1.22)**	1.05(0.98–1.12)	1.05(0.99–1.12)	0.11(-0.21-0.43)
Post intervention period	1.17(1.09–1.26)***	1.14(1.06–1.22)***	1.06(1.00-1.14)*	1.06(1.00-1.13)*	0.32(0.04–0.60)*
Month	1.00(1.00–1.00)**	1.00(0.99-1.00)	1.00(1.00-1.01)***	1.00(1.00-1.01)***	

Note: IRR – Incidence Rate Ratio; aIRR – adjusted Incidence Rate Ratio; ATET – average treatment effect on the treated; *p < 0.05; **p < 0.01;

The unadjusted and adjusted logistic regression results of each QI in the intervention compared to the control site during the baseline and intervention are presented in Table 5. The odds of vaginal birth were higher during the baseline at the intervention site but insignificant in the adjusted model. During the intervention, the odds of vaginal birth significantly remained higher in the experiment site compared to the control site. The odds of using a clean delivery pack were lower at the intervention site but only significant during the intervention period. The report that something was applied on the stump after the umbilical cord was cut was significantly lower in the experiment compared to the control site before and during the intervention. Baby placed on the belly or breast before delivery of the placenta was significantly higher in the experiment site at baseline, but only when no factor was adjusted; and the odds became significantly lower during the intervention period.

**Table 5:** Unadjusted and adjusted Logistic regression of Intrapartum Care Quality Indicators in the intervention site versus control site(ref)

Indicator	Baseline period		Intervention period		ATET(95% CI)
	OR(95%CI)	aOR(95% CI)	OR(95%CI)	aOR(95% CI)	
Assistance during delivery by					
Midwife/doctor vs doctor only	2.86(0.72–11.38)	2.35(0.49–11.14)	0.77(0.39–1.52)	0.64(0.31–1.29)	-0.003(-0.042-0.363)
Vaginal birth vs CS	2.60(1.19–5.70)*	2.54(0.64–9.97)	1.75(1.23–2.50)**	1.58(1.03–2.44)*	0.121(0.023–0.218)*
Clean delivery pack used vs no	0.98(0.06–16.08)	0.20(0.00-6.64)	0.06(0.01–0.28)***	0.05(0.01–0.23)***	-0.075(-0.102- -0.048)***
Something applied on the stump after umbilical cord was cut vs no	0.32(0.14–0.72)**	0.12(0.04–0.36)***	0.06(0.04–0.11)***	0.04(0.02–0.07)***	-0.415(-0.479- -0.351)***
Baby dried before placenta was delivered vs no	1.49(0.69–3.22)	0.89(0.33–2.35)	1.03(0.75–1.41)	0.94(0.67–1.31)	-0.067(-0.160- 0.024)
Baby was placed on belly/breast before delivery of placenta vs no	2.61(1.03–6.64)*	1.44(0.45–4.54)	0.88(0.62–1.25)	0.64(0.43–0.95)*	0.005(-0.109-0.119)
Baby bathed first time 24 hours + vs < 24 hours	0.35(0.16–0.75)**	0.29(0.11–0.76)*	0.33(0.24–0.47)***	0.40(0.28–0.57)***	-0.167(-0.257 -0.076)***
Given verbal/or printed information on baby care vs no	0.84(0.40–1.75)	1.32(0.54–3.23)	0.70(0.49-1.00)*	0.79(0.54–1.15)	-0.105(-0.197- -0.013)*
Well attended to during labour vs no	0.98(0.26–3.58)	0.33(0.05–2.02)	2.59(1.62–4.14)***	2.24(1.37–3.64)**	0.095(0.030–0.159)*
No episiotomy during labour vs yes	2.32(1.11–4.84)*	2.95(1.22–7.09)*	0.61(0.42–0.88)*	0.67(0.45–0.99)*	0.016(-0.080- 0.112)
**Sub-population analysis**					
Happy with how health worker helped to reduce pain during labour vs no	0.79(0.38–1.63)	0.80(0.33–1.93)	4.91(2.95–8.20)***	4.04(2.32–7.01)***	0.194(0.084- 0.304)**
Episiotomy - Cut promptly repaired vs no	Omitted	omitted	20.32(6.60-62-55)***	18.16(5.33–61.78)***	0.247(0.048- 0.446)*

Note: Due to small values, analysis by the segmented 7 periods could not be done. Omitted because no in the intervention site was zero during the baseline. OR – Odds Ratio; aOR – adjusted Odds ratio; ATET – average treatment effect on the treated; *p < 0.05; **p < 0.01; ***p < 0.001

Relative to the control site, bathing the baby for the first time less than 24 hours after birth was significantly less in the experiment site both at baseline and intervention period. The likelihood of being given verbal/or printed information on how to care for a baby was significantly lower in the experiment site, but only in the unadjusted bivariate model. Compared to the control site, the report of being well attended during labour was insignificantly lower in the experiment site at baseline, but during the intervention period, the odds became significantly higher even after controlling the effect of other factors (aOR 2.24 CI: 1.37–3.64).

The odds of not having episiotomy were significantly higher in the experiment site than in the control site at baseline, it significantly decreased during the intervention period. Among the respondents who had an episiotomy, the odds of reporting that the cut was repaired promptly was 18.16 times more likely in the experiment than in the control site (aOR 18.16 CI:5.33–61.78). Among the 73% who reported severe pain during labour, the probability of reporting that she was happy with how the health worker helped to reduce the pain was insignificantly lower in the experiment than in the control site at baseline, but the odds significantly increased in the experiment site during the intervention period (aOR 4.40 CI:2.32–7.01).

The ATET was significantly positive for vaginal versus caesarean section birth, well attended during labour, happy with how health workers helped to reduce pain during labour, and prompt repair of episiotomy. The ATET for lower likelihood of bathing a baby for the first time 24 hours plus was also significant.

## Discussion

The study was designed to investigate the effectiveness of a set of interventions in improving the quality of intrapartum care in Nigerian referral hospitals. The interventions which were co-designed and implemented by healthcare providers and administrators addressed multiple challenges associated with inadequate delivery of intrapartum care in the hospitals. No adverse or simultaneous intervention took place in the experiment and control hospitals during the period of this study. Contrary to expectation, the results showed that the aggregate assessment of the quality of intrapartum care was higher in the control sites as compared to the intervention sites in both adjusted and unadjusted regression. However, an analysis of the trend over three months showed significant improvement in the intervention site at the mid and end periods of the intervention.

In this study, we assessed ten quality of intrapartum care indicators from the WHO recommendations for positive childbirth experience[9] as an aggregate care quality measure. Despite the lack of effectiveness of the interventions in improving the quality of care overall, some components of the intrapartum care indicators performed better in the intervention sites as compared to the control sites. To date, several published studies have investigated the quality of intrapartum care in different regions of Nigeria [7, 22–25], but none has focused on interventions that address the delivery of quality intrapartum care. The results of this study, therefore, have implications for further research to provide evidence on ways to improve the quality of intrapartum care and reduce maternal mortality in Nigeria.

Overall, the interventions showed 6% increase in overall quality of care and demonstrated beneficial effects in five principal areas. These include (1) perceptions by women that they were well attended in labour; (2) higher rates of vaginal delivery as compared to caesarean delivery; (3) prompt repair of episiotomies; (4) better pain management in labour; (5) delayed bath of the baby after delivery.

The mode of delivery showed considerable improvement in the intervention sites as compared to the control sites. While the WHO recommends worldwide caesarean section rates not exceed 15% , published studies conducted in referral hospitals in Nigeria have repeatedly shown that cesarean section rates are over this average [26, 27]. Thus, intervention training emphasized the need to use caesarean sections only for acceptable indications, to manage labour in ways for early detection of labour challenges, and to apply appropriate remediating measures. Understandably, the vaginal delivery rate was higher in the intervention sites compared to the control sites, and the caesarean section rates were significantly lower. This underscores the effectiveness of the intervention in reducing caesarean section rates in referral hospitals.

The intervention also had effects on the use of episiotomy in the hospitals. Why the WHO now does not recommend routine use of episiotomy [9], several published studies in Nigeria have continued to show high prevalence rates of routine episiotomy [28–30]. Thus, during the training and development of labour management protocols, we emphasized the non-use of routine episiotomy and demonstrated ways to ensure safe delivery without episiotomy. However, surprisingly, the intervention results showed higher rates of episiotomy in the intervention sites compared to the control sites. This may suggest episiotomy was often needed to prevent excessive use of caesarean, considering complex labour cases are often referred to these hospitals. Alternatively, reduced caesarean at intervention sites may indicate a benefit of the intervention training as women who had an episiotomy reported early and prompt repair compared to control sites.

A major outcome of the intervention was its beneficial effects in improving women’s experiences of labour pains. Up to 73% of the women in both sites reported experiences of severe pains during labour. However, the report showed that women in the intervention sites were more likely to report that they were happier with the ways health workers managed labour pains as compared to the control sites. Overall, the women in the intervention sites reported higher odds of not being satisfied with how the health workers helped to reduce pain during labour at baseline, but significantly higher odds during the intervention. This indicates that women in the intervention sites were progressively more satisfied with intrapartum care. A previous assessment of overall satisfaction with maternal care after the intervention shows women in the intervention sites were 54% more likely to be satisfied than those in the control site[17]. We conjecture this to be attributable to the training provided to all staff at the intervention sites, which emphasized pain management, and the various elements of the delivery of respectful intrapartum care. A training-based intervention in India also reported improvement in providers’ adherence to recommended practices during childbirth [31]. However, the results of the high rate of painful labour in all sites suggest a need for a more proactive and effective approach to managing labour in Nigeria’s referral hospitals.

Another positive outcome of the intervention was the report of delayed bath of the babies after delivery. The WHO recommends that babies be bathed not sooner than 24 h after delivery [9] in order to keep the babies warm. The results of this study showed that women in the intervention, compared to those in the control sites, were less likely to bathe their babies within the first 24 h. This is possibly attributable to the training provided to health workers in the intervention sites.

In contrast to the positive outcomes specified above, the results of this analysis showed that the intervention had no or limited effects in ameliorating at least five signal functions. These include (1) reports of the use of clean delivery packs being lower in the intervention sites as compared to the control sites; (2) women reporting that something was applied to the umbilical cord after the birth of the baby lower in the intervention sites; (3) baby placed in the belly with odds lower in the intervention sites; (4) the lack of effects of the intervention in reducing the incidence of routine episiotomy, and (5) reports by women that verbal and written information were less likely to be provided in the intervention sites as compared to the control sites.

These limited effects of the intervention may be due to the imperfect implementation of some of the intervention activities or poor integration of the activities into care provision in the intervention hospitals. For example, women who did not receive antenatal care in the hospitals but were referred during labour did not receive the training and information given to women in antenatal clinics and weekly health talks. It is important to ensure all women receive all components of quality care regardless of the timing of their admission into the hospital care system [9].

Recall bias may not have accounted for some of these differences as women in intervention and control sites are equally likely to experience the same level of bias, given that they were recruited and interviewed during the same period.

### Study strengths and limitations

To the best of our knowledge, this study is one of a few studies that address improving the quality of intrapartum care in sub-Saharan Africa. Given the high rate of maternal mortality in the continent, it is surprising that many such interventions have previously not been conducted to improve the quality of care in labour, a period when women are most likely to die during childbirth. The adoption of a model based on initial formative research followed by co-design and implementation of the research by responsible health workers makes this approach particularly appealing and sustainable. The formative research, in particular, would help to identify areas of knowledge and skills gaps by health providers in the management of labour, which can then be rectified by specific training during the intervention.

This study is limited by the fact that it was restricted to referral hospitals, rather than primary health centres that provide normal labour and delivery care. However, in view of the resource deficits currently prevailing in Nigeria’s primary health care system [32], we felt it more appropriate and logical to conduct the study in referral facilities to provide lessons to be used at different levels of the health care system. This approach would also help the supervisory roles that referral hospitals provide for primary health centres in the country. Other limitations include that quasi-experiments do not provide evidence as strong as those from randomized controlled trial. Also, the number of participants from each arm is not large; this limits the extent of generalization of the findings. Finally, the measure of quality of intrapartum care in this study is subjective reports of women based on their experience. Using both subjective and objective measures of quality of intrapartum care, and including supply-side perspectives would provide a more holistic view of the quality of intrapartum care in the hospitals.

## Conclusions

In conclusion, the multiple interventions that address various components of the quality of intrapartum care in Nigeria’s referral hospitals demonstrated some effectiveness. The interventions improved five of ten quality indicators. We believe that the approach to developing interventions based on formative research is important, but a process of integrating the implementation activities with the normal maternal health delivery processes in the hospitals will enhance the effectiveness of this approach.

## Data Availability

The dataset analyzed for the current study is available from the corresponding author on request.

## List of Abbreviations

WHO: World Health Organization
NCTR: Nigeria Clinical Trials Registry
ISRCTN: International Standard Randomised Controlled Trial Number
QI: Quality Indicators
CS: Caesarean Section
CAPI: Computer-assisted Personal Interview
IRR: Incidence Risk Ratio
aIRR: Adjusted Incidence Risk Ratio
CI: Confidence Interval

## References

[1] Akaba GO, Nnodu OE, Ryan N, Peprah E, Agida TE, Anumba DO (2021). Applying the WHO ICD-MM classification system to maternal deaths in a tertiary hospital in Nigeria: a retrospective analysis from 2014–2018. PLoS ONE.

[2] Piane GM (2019). Maternal mortality in Nigeria: a literature review. World Med Health Policy.

[3] Tukur J, Lavin T, Adanikin A, Abdussalam M, Bankole K, Ekott MI (2022). Quality and outcomes of maternal and perinatal care for 76,563 pregnancies reported in a nationwide network of nigerian referral-level hospitals. EClinicalMedicine.

[4] National Population Commission (NPC) (2019). [Nigeria] and ICF. Nigeria Demographic and Health Survey 2018.

[5] Ntoimo LFC, Okonofua FE, Ekwo C, Solanke TO, Igboin B, Imongan W et al. Why women utilize traditional rather than skilled birth attendants for maternity care in rural Nigeria: Implications for policies and programs. Midwifery [Internet]. 2022;104:103158. Available from: https://www.sciencedirect.com/science/article/pii/S0266613821002382. 10.1016/j.midw.2021.103158 34700126

[6] Titaley CR, Hunter CL, Dibley MJ, Heywood P (2010). Why do some women still prefer traditional birth attendants and home delivery?: a qualitative study on delivery care services in West Java Province, Indonesia. BMC Pregnancy Childbirth.

[7] Bohren MA, Vogel JP, Tunçalp Ö, Fawole B, Titiloye MA, Olutayo AO (2017). Mistreatment of women during childbirth in Abuja, Nigeria: a qualitative study on perceptions and experiences of women and healthcare providers. Reproductive Health.

[8] Okonofua F, Ogu R, Agholor K, Okike O, Abdus-Salam R, Gana M (2017). Qualitative assessment of women’s satisfaction with maternal health care in referral hospitals in Nigeria. Reproductive health.

[9] World Health Organization (2018). WHO recommendations: intrapartum care for a positive childbirth experience.

[10] Ishola G, Ajala AO, Ugwa E, Alobo G, Okoli U, Rawlins B (2020). Quality of labour and uncomplicated delivery care: a formative assessment of selected health facilities in Ebonyi and Kogi States, Nigeria. Afr J Reprod Health.

[11] Okonofua FE, Ogu RN, Agholor K, Galadanci H, Abdus-Salam A, Okike O (2016). An intervention to improve the management of obstetric emergencies and prevent maternal and perinatal deaths in Nigeria: a quasi-experimental research design.

[12] Okonofua FE, Ogu R, Imongan W. Nigeria Clinical Trials Registry, Federal Ministry of Health, Abuja, Nigeria. 2016. Assessing the impact of an intervention to improve the quality of emergency obstetric care on maternal and perinatal mortality H9-TSA-282. Available from: https://nctr.nhrec.net/viewTrials.php?TID=91540209.

[13] Okonofua FE, Randawa A, Ogu RN, Agholor K, Okike O, Abdus-Salam A (2017). Views of Senior Health Personnel about Quality of Emergency Obstetric Care: a qualitative study in Nigeria. PLoS ONE.

[14] Okonofua FE, Ogu RN, Ntoimo LF, Gana M, Okike ON, Durodola A (2018). Where do delays occur when women receive antenatal care? A client flow multisite study in four health facilities in Nigeria. Ghana Med J.

[15] Okonofua FE, Ekezue B, Ntoimo LFC, Ekwo C, Ohenhen V, Agholor K (2022). Effects of multifaceted interventions to prevent and manage primary postpartum haemorrhage in referral hospitals: a quasi-experimental study in Nigeria. BMJ global health.

[16] Okonofua FE, Ntoimo LFC, Ekezue B, Ohenhen V, Agholor K, Gana M et al. Outcome of multifaceted interventions for improving the quality of antenatal care in Nigerian referral hospitals. Reproductive Health [Internet]. 2020;17(1):170. Available from: 10.1186/s12978-020-00997-6. 10.1186/s12978-020-00997-6 PMC7641810 33148284

[17] Okonofua FE, Ntoimo LFC, Ekezue BF, Ohenhen V, Agholor K, Igboin B et al. Outcomes of a multifaceted intervention to improve maternal satisfaction with care in secondary hospitals in Nigeria. Global Health Action [Internet]. 2020 Dec 31 [cited 2021 Aug 3];13(1):1856470. Available from: 10.1080/16549716.2020.1856470. 10.1080/16549716.2020.1856470 PMC7751393 33334274

[18] Okonofua F, Ekezue BF, Ntoimo LF, Ohenhen V, Agholor K, Imongan W et al. Outcomes of a multifaceted intervention to prevent eclampsia and eclampsia-related deaths in Nigerian referral facilities. International Health [Internet]. 2023 Jun 30 [cited 2023 Jul 7];ihad044. Available from: 10.1093/inthealth/ihad044. 10.1093/inthealth/ihad044 PMC11062200 37386659

[19] Aikpitanyi J, Ohenhen V, Ugbodaga P, Ojemhen B, Omo-Omorodion BI, Ntoimo LF (2019). Maternal death review and surveillance: the case of Central Hospital, Benin City, Nigeria. PLoS ONE.

[20] Yamane T, Statistics. An introductory analysis. 2nd Edition. New York; 1967.

[21] World Bank, Federal Ministry of Health, National Bureau of Statistics (NBS). Health Results Based Financing Nigeria 2017 Exit Interview for Antenatal care Visit. 2017.

[22] Bohren MA, Vogel JP, Tunçalp Ö, Fawole B, Titiloye MA, Olutayo AO (2016). By slapping their laps, the patient will know that you truly care for her”: a qualitative study on social norms and acceptability of the mistreatment of women during childbirth in Abuja. Nigeria SSM-population health.

[23] Chukwuma A, Mbachu C, Cohen J, Bossert T, McConnell M (2017). Once the delivery is done, they have finished”: a qualitative study of perspectives on postnatal care referrals by traditional birth attendants in Ebonyi state, Nigeria. BMC Pregnancy Childbirth.

[24] Esienumoh EE, Akpabio II, Etowa JB, Allotey J, Waterman H (2016). Cultural diversity in childbirth practices of a rural community in Southern Nigeria. J Preg Child Health.

[25] Ojong I, Ndukaku N, Kalu U, Blessing O (2019). Predictors of women’s perception of Intrapartum Care in a secondary health facility in Calabar Metropolis, Cross River State, Nigeria. J Preg Child Health.

[26] Allagoa DO, Oriji PC, Tekenah ES, Obagah L, Ohaeri OS, Mbah KM (2021). Caesarean section in a Tertiary Hospital in South-South, Nigeria: a 3-year review. Eur J Med Health Sci.

[27] Osegi N, Makinde OI (2020). Towards optimizing caesarean section: a five-year review of caesarean sections at a Southern Nigeria hospital. Int J Reprod Contracept Obstet Gynecol.

[28] Awoyesuku PA, John DH, Altraide BO (2020). Prevalence and predictors of episiotomy and perineal tear at a tertiary hospital in Port-Harcourt, Nigeria. Int J Reprod Contracept Obstet Gynecol.

[29] Hembah-Hilekaan S, Ojabo A, Audu O, Onche P, Maanongun M (2018). Prevalence of episiotomy and perineal lacerations in a University Teaching Hospital, North-Central Nigeria. J BioMedical Res Clin Pract.

[30] Ononuju CN, Ogu RN, Nyengidiki TK, Onwubuariri MI, Amadi SC, Ezeaku EC (2020). Review of episiotomy and the effect of its risk factors on postepisiotomy complications at the University of Port Harcourt Teaching Hospital. Nigerian Med Journal: J Nigeria Med Association.

[31] Singh S, Kannuri NK, Mishra A, Gaikwad L, Shukla R, Tyagi M (2022). Effect of WHO-SCC based intra-department mentoring program on quality of intrapartum care in public sector secondary hospitals in Andhra Pradesh, India: pre-post mixed methods evaluation. PLOS Global Public Health.

[32] Hanson K, Brikci N, Erlangga D, Alebachew A, De Allegri M, Balabanova D (2022). The Lancet Global Health Commission on financing primary health care: putting people at the centre. The Lancet Global Health.

